# Serum immunoglobulins in 28 adults with autoimmune sensorineural hearing loss: increased prevalence of subnormal immunoglobulin G1 and immunoglobulin G3

**DOI:** 10.1186/s12865-014-0043-2

**Published:** 2014-10-22

**Authors:** Luigi F Bertoli, Dennis G Pappas, J Clayborn Barton, James C Barton

**Affiliations:** Brookwood Medical Center, Birmingham, AL USA; Brookwood Biomedical, Birmingham, AL USA; Pappas Ear Clinic, Birmingham, AL USA; Southern Iron Disorders Center, Birmingham, AL USA; Department of Medicine, University of Alabama at Birmingham, Birmingham, AL USA

**Keywords:** For indexing, Autoimmune sensorineural hearing loss, Common variable immunodeficiency (CVID), IgG, IgG subclass deficiency (IgGSD), IgG1, IgG3

## Abstract

**Background:**

Our informal observations suggested that some patients with acute sensorineural hearing loss (ASNHL) have subnormal serum immunoglobulin (Ig) levels. We evaluated 28 consecutive adults (18 men, 10 women) at ASNHL diagnosis using: antibodies to 68 kD protein, 30 kD protein, and type II collagen; and serum total IgG, IgG subclasses, total IgA, and IgM. Reference ranges for Ig levels were mean ± 2 SD. We compared prevalences of subnormal IgG subclasses to those in 275 healthy European adults in previous reports. We also reviewed charts of consecutive adult index patients with primary Ig deficiency (35 common variable immunodeficiency, 406 IgG subclass deficiency) to identify other patients with probable ASHNL.

**Results:**

Mean age was 53 ± 10 (SD) y. Six patients (21.4%) had other autoimmunity manifestations. Antibodies to 68 kD protein, 30 kD protein, and type II collagen were detected in 21.4% (6/28), 21.1% (4/19) and 18.8% (3/16), respectively. Three patients (10.7%) had subnormal IgG1, six (21.4%) had subnormal IgG3, and four (14.3%) had subnormal IgG1 and IgG3. Some had subnormal IgG2, IgG4, IgA, and IgM (n = 1, 2, 3, and 1, respectively). Prevalences of subnormal IgG1 or IgG3 were greater in ASNHL patients (25.0% and 35.7%) than 275 controls (2.1% and 3.3%), respectively (p < 0.0001, each comparison). Relative risks of subnormal IgG1 and IgG3 in ASNHL were 11.5 [95% CI: 4.1, 31.7] and 10.9 [4.8, 25.6], respectively. Hearing improved after initial therapy in 17 patients (60.7%). Multiple regressions on Ig levels revealed no significant associations with other available variables. Logistic regressions on initial therapy response revealed a positive association with men (p = 0.0392) and a negative association with IgA (p = 0.0274). Our estimated prevalence of probable ASNHL in 35 patients with common variable immunodeficiency during a follow-up interval of 8 ± 4 y was 0% [95% CI: 0, 12.3]). Prevalence of probable ASNHL in 406 patients with IgG subclass deficiency during the same interval was 0.74% [0.19, 2.33].

**Conclusions:**

Serum levels of IgG1 or IgG3 were subnormal in 46.4% of 28 patients with ASNHL. Among adults who present with primary Ig deficiency, some may have or later develop ASNHL.

## Background

Autoimmune sensorineural hearing loss (ASNHL), also called autoimmune inner ear disease or autoimmune hearing loss, is characterized by rapidly progressive sensorineural hearing loss not explained by other defined causes [1-3]. Inner ear antigens that have been proposed as targets of putative deleterious autoantibodies in ASNHL include, but are not limited to, 68 kD protein (often called heat-shock protein) [4-6], 30 kD protein (often called myelin Protein 0 or P0) [7,8], type II collagen [9], choline transporter-like protein 2 [10-12], cochlin [13], and inner ear supporting cell antigen [14]. Many patients with ASNHL experience rapid improvement of hearing with initial immunosuppressive therapy such as corticosteroids (systemic or intratympanic) [15], methotrexate [16], or cyclophosphamide [1]. Analysis of a large medical and pharmaceutical claims database indicated that the incidence of sudden sensorineural hearing loss in the U.S. is 27 per 100,000 per year [17].

In persons with diverse autoimmune disorders, the prevalence of immunoglobulin (Ig) phenotypes typical of common variable immunodeficiency (CVID) or IgG subclass deficiency (IgGSD) is increased [18-20]. Thus, we reviewed charts of 28 consecutive white adults referred to a single practice for immunologic evaluation at diagnosis of ASNHL. We measured serum levels of total IgG, IgG subclasses, IgA, and IgM at diagnosis and estimated the prevalence of subnormal Ig values in this cohort. We also estimated the prevalence of ASNHL in 441 adult white index patients with CVID/IgGSD who presented with increased susceptibility to upper and lower respiratory tract infections. We discuss the pertinence of our findings to the evaluation, management, and pathogenesis of autoimmune hearing loss.

We compared the prevalences of subnormal IgG subclass levels in the present 28 patients with ASNHL with those in 275 healthy adults in three previous reports [21-23]. We selected these reports because: a) abnormal IgG subclass levels were defined as values more than 2 SD from the mean, as in the present study; b) distinguishing subjects with single subclass deficiency from those with deficiency of two or more subclasses was possible; and c) numbers of control subjects were at least as great in the present ASNHL cohort [21-23]. We also estimated the prevalence of ASNHL in the 441 index patients with CVID/IgGSD.

## Methods

### Patient selection

The performance of this work was approved by the Institutional Review Board of Brookwood Medical Center. Written informed consent of study subjects was not required because the present report is based solely on retrieval and analysis of observations made as part of routine medical care delivery and does not include personal identifier information. All patients were white adults referred to a single hematology and medical oncology practice. We evaluated two groups of patients. The first cohort comprised 28 consecutive unrelated patients referred by a single otolaryngology practice during the interval 1998–2014 for immunological assessment at diagnosis of ASNHL, including quantification of serum Ig levels. The second cohort, 441 unrelated patients, were referred during the interval 1998–2012 for evaluation and management of hypogammaglobulinemia associated with increased frequency or severity of upper or lower respiratory tract infections poorly controlled by antibiotic therapy and diagnosed to have CVID (n = 35) or IgGSD (n = 406). All patients resided in Alabama.

### Definition of ASNHL

ASNHL is defined herein as rapidly progressive bilateral sensorineural hearing loss not explained by other defined causes [1-3]. Inclusion or exclusion in this ASNHL case series was not based on the presence or absence of antibodies reactive with 68 kD protein, 30 kD protein, or type II collagen or on response to initial immunosuppressive therapy. The description of ASNHL patients is provided with regard to unilateral/bilateral hearing loss and the audiometric findings. Specifically, the Gardner-Robertson Hearing Scale is utilized and referenced for both the better and worse hearing ear.

### Definition of CVID and IgGSD

We defined probable CVID in accordance with the criteria of the Pan-American Group for Immunodeficiency and the European Society for Immunodeficiency [24]. In adults, these criteria include either men or women with decreased levels of serum IgG and IgA at least 2 SD below the mean; absent isohemagglutinins or poor response to vaccines; and exclusion of other defined causes of hypogammaglobulinemia [24]. IgGSD was defined as deficiency of one or more IgG subclasses (IgG1-3) at least 2 SD below the mean in the presence of normal total serum IgG levels, with or without IgA deficiency [25].

Each patient diagnosed to have CVID or IgGSD was tested for IgG levels specific for polysaccharide antigens of one or more common serotypes of *Streptococcus pneumoniae*. We designated the first persons in respective families diagnosed to have CVID/IgGSD as index patients. We performed a manual review of medical charts and a computerized review of electronic billing records to identify CVID/IgSD index patients who also had histories or manifestations suggesting that they had ASNHL.

### Patient exclusions

ASNHL cohort. Patients with defined causes of hearing loss other than ASNHL were excluded by history, initial otologic and audiometric examinations, or diagnostic abnormalities of immunologic and other assessments. We excluded patients with clinical characteristics of Ménière disease or immunologic abnormalities typical of Wegener granulomatosis.

CVID/IgGSD cohort. We excluded patients from this cohort who were referred primarily for hearing loss evaluation. We excluded subjects with isolated deficiencies of IgA or IgM, normal immunoglobulin levels with deficiency of selective IgG specificity for *Streptococcus pneumoniae* polysaccharide antigen(s) serotypes; or hypogammaglobulinemia attributed to B-cell neoplasms, organ transplantation, immunosuppressive therapy, or increased immunoglobulin loss. We excluded patients with either monoclonal gammopathy of uncertain etiology, polyspecific gammopathy, or diagnosis of HIV infection.

We excluded patients of African American descent because: a) mean serum concentrations of immunoglobulins are often greater in adults of sub-Saharan African descent than in white adults [26,27]; and b) persons of sub-Saharan descent occur infrequently in series of CVID or IgGSD patients [28]. During the study interval in which we diagnosed 441 white patients with CVID/IgGSD, we also evaluated and diagnosed two African Americans who met the same diagnostic criteria. Thus, only 2/443 CVID/IgGSD patients were African American (0.0045 [95% CI: 0.0001, 0.0174]; Wald method).

### Laboratory methods

Assessments of anti-nuclear antibody (ANA), rheumatoid factor, cytoplasmic anti-neutrophil cytoplasmic antibodies (c-ANCA), perinuclear anti-neutrophil cytoplasmic antibodies (p-ANCA), anti-cardiolipin antibodies (IgG, IgA, and IgM specificities), and serum protein immunoelectrophoreses (SIEP) were performed using routine methods. Testing for serum levels of anti-68 kD protein, anti-30 kD protein, and anti-type II collagen antibodies was performed by IMMCO Diagnostics, Inc. (Buffalo, NY). Positivity for type II collagen antibody was defined as >25 EU/mL.

Serum Ig levels were measured at diagnosis in patients with ASNHL or before initiation of IgG replacement therapy in patients diagnosed to have CVID/IgGSD. Serum concentrations of total IgG, IgG subclasses, IgA, and IgM were measured using standard automated methods. We defined mean ± 2 SD as the normal or reference range for all Ig measurements [18,21-23]. Reference ranges for serum immunoglobulin concentrations are: total IgG 7.00-16.00 g/L; IgG1 4.22-12.92 g/L; IgG2 1.17-7.47 g/L; IgG3 0.41-12.9 g/L; IgG4 0.01-2.91 g/L; total IgA 910–4140 mg/L; and IgM 400–2300 mg/L. Subnormal serum Ig levels were defined as concentrations below the corresponding lower reference limit.

### Statistics

We performed initial logistic regressions on response to initial immunosuppressive therapy (dichotomous variable) in patients with ASNHL using these independent variables: age; sex; serum levels of IgG subclasses, IgA, and IgM; and positivity for anti-68 kD protein, anti-30 kD protein, and anti-type II collagen antibodies (as dichotomous variables). We excluded variables with values of p >0.1500 in initial regressions from the final regression models.

Analyses were performed with Excel 2000® (Microsoft Corp., Redmond, WA) and GB-Stat® (v. 10.0, 2003, Dynamic Microsystems, Inc., Silver Spring, MD). Descriptive data are displayed as enumerations, percentages, or mean ± 1 standard deviation (SD). Prevalence or frequency values were compared using chi-square analysis or Fisher exact test (one-tail), as appropriate. We used the Wald method to compute confidence intervals of proportions. Prevalence estimates computed with continuity corrections are expressed as percentage [95% confidence interval, CI]. We computed relative risks (RR) by comparing the present data with appropriate control observations. Multiple or logistic regressions were performed on some independent variables, as appropriate, to identify their associations with other variables. A value of *p* <0.05 was defined as significant.

## Results

### General characteristics

There were 28 patients (18 men; 10 women). Mean age at diagnosis was 53 ± 10 y. Results of pre-treatment hearing tests were available for review in 25 of the 28 patients (89.3%). All 25 patients had bilateral asymmetric sensorineural hearing loss. According to the Gardner-Robertson Hearing Scale [29], the better hearing ear for each patient was classified as Grade I (Good) in 15 patients (60%), Grade II (Serviceable) in 7 patients (28%), Grade III (Non-serviceable) in 3 patients (12%), and Grade IV (Poor) and Grade V (Deaf) in no patients. The worse hearing ear for each patient was classified as Grade I in 1 patient (4%), Grade II in 9 patients (36%), Grade III in 11 patients (44%), Grade IV in no patients, and Grade V in 4 patients (16%). An accurate determination of duration of symptoms was not available for most patients. No patient had features of or was diagnosed to have Cogan syndrome.

### Autoimmune manifestations

We tabulated reports of autoimmune manifestations in each patient. One patient each had a history of “acute” rheumatoid arthritis, seronegative rheumatoid arthritis, Reiter’s syndrome, ulcerative colitis, and elevated ANA (titer 1:320), respectively (6/28; 21.4%). There was chronic activity of seronegative rheumatoid arthritis in one man, intermittent activity of uveitis due to Reiter’s syndrome in another man, and intermittent activity of ulcerative colitis in a woman. Three of ten women (33.3%) had hypothyroidism (etiology not specified). Another woman (10.0%) had hypopituitarism.

### Positivity for putative inner ear autoantibodies

Each of the present 28 patients (100%) was tested for positivity for anti-68 kD protein. Some patients were also tested for other antibody specificities. Eight patients were tested for a single antibody specificity; two of them (25.0%) had a positive antibody test result. Five patients were tested for two antibody specificities; three of them (60.0%) were positive for a single antibody specificity. Fifteen patients were tested for all three specificities. Five of the 15 patients were positive for a single antibody specificity (33.3%) and the sixth patient was positive for all three specificities. A Pearson’s chi-square test revealed that these proportions across the three antibody specificities tested did not differ significantly (p = 0.4522).

Anti-68 kD protein antibody was detected in 6 of 28 patients (21.4%; [95% CI: 9.0, 41.5]). Anti-30 kD protein antibody was detected in 4 of 19 patients (21.1%; [7.0, 46.1]). Anti-type II collagen antibody was detected in 3 of 16 patients (18.8%; [5.0, 46.3]).

### Miscellaneous test results

The initial assessment in each patient included complete blood count, ANA, rheumatoid factor, anti-leukocyte antibodies (c-ANCA; p-ANCA), and anti-cardiolipin antibodies. Some patients underwent testing for syphilis or human immunodeficiency virus-1 infection, hematologic malignancy, heritable disorders, or abnormalities detectable on magnetic resonance imaging, as appropriate. No patient had significant abnormalities detected by these tests (results not shown). SIEP did not reveal a monoclonal immunoglobulin in any patient.

### Serum Ig levels

At diagnosis, mean Ig levels (range) in 28 patients were: total IgG 8.67 ± 2.20 g/L [95% CI: 7.82, 9.52]; IgG1 5.01 ± 1.62 g/L [4.36, 5.64]; IgG2 2.87 ± 1.11 g/L [2.43, 3.31]; IgG3 0.53 ± 0.28 g/L [0.42, 0.64]; and IgG4 0.25 ± 0.19 g/L [0.17, 0.32]. Mean total IgA level was 1790 ± 730 mg/L [1500, 2070]. Mean IgM level was 1230 ± 930 mg/L [870, 1590]. Subnormal serum levels of total IgG were detected in five patients (17.9%). Three patients (10.7%) had subnormal IgG1, six (21.4%) had subnormal IgG3, and four (14.3%) had subnormal IgG1 and IgG3. Subnormal serum levels of either IgG1 or IgG3 were observed in nine men (50.0%) and four women (40.0%). Two patients had subnormal serum levels of IgG4: one of the two patients had a subnormal serum level of IgG1 and the other patient had subnormal serum levels of IgG1, IgG2, and IgG3. Altogether, 13 patients (46.4%) had subnormal serum levels of one or more IgG subclass (es).

Three of six patients (50.0%) who were positive for anti-68 kD protein antibody also had subnormal serum levels of IgG1 or IgG3. Ten of 22 patients (45.5%) who were negative for anti-68 kD protein antibody had subnormal serum levels of IgG1 or IgG3. The difference between these proportions was not significant (50.0% vs. 45.5%; p = 0.6005).

Subnormal serum levels of total IgA were observed in three patients (740, 750, and 780 mg/L, respectively). One patient had a subnormal serum level of IgM (150 mg/L). Mild elevation of serum levels of IgM were observed in four patients (2510, 2580, 2850, and 4090 mg/L, respectively). No patient had an increased serum level of total IgG, a IgG subclass, or total IgA. No patient had evidence of a monoclonal protein by SIEP criteria.

### Prevalence and RR of subnormal Ig levels in ASNHL patients and controls

We combined previous observations on IgG subclass levels in three groups of healthy subjects previously reported (125 Irish blood donors; 68 German blood donors; 82 English women) to a create an aggregate group of 275 controls [21-23] (Table 1). The prevalences of subnormal serum IgG1 and IgG3 levels in 28 patients with ASNHL were significantly higher than corresponding levels in the 275 control subjects (Table 1). The proportions of normal controls who had subnormal IgG1 and IgG3 levels (2.1% and 3.3%, respectively) are consistent with the 2.5% of values predicted to occur below 2 SD from the mean (Table 1). The prevalence of subnormal serum IgG2 and IgG4 levels in 28 patients with ASNHL did not differ significantly from those in 275 control subjects (Table 1).

**Table 1:** **Subnormal serum IgG subclass levels in adults** ^**a**^

**Cohort**	**Subnormal Ig level (n)** ^**b**^	**Prevalence [95% CI]; value of p** ^**c**^	**Relative risk [95% CI]; value of p** ^**c**^
28 ASNHL patients	IgG1 (7)	25.0 [11.4, 45.2]; <0.0001	11.5 [4.1, 31.7]; <0.0001
	IgG2 (1)	3.6 [0.2, 20.2]; 0.3863	2.5 [0.3, 21.2]; 0.4143
	IgG3 (8)	35.7 [9.3, 55.9]; <0.0001	8.7 [3.7, 20.8]; <0.0001
	IgG4 (2)	7.1 [1.3, 25.0]; 0.1978	3.0 [0.7, 13.8]; 0.1561
275 healthy controls	IgG1 (6)	2.1 [0.9, 4.9]	-
	IgG2 (4)	1.5 [0.5, 3.9]	-
	IgG3 (9)	3.3 [1.6, 6.3]	-
	IgG4 (7)	2.6 [1.1, 5.4]	-

^a^Reference ranges for serum IgG subclass levels were mean ± 2 SD for age. Controls included: 125 healthy Irish adult blood donors (68 men, 57 women; ages 48 ± 16 (range 22–81 y); 82 healthy English women (mean 57 y; range 22–90 y); and 68 healthy German blood donors (36 men, 32 women; ages 20–61 years) [21-23].

^b^Subnormal serum IgG subclass levels were reported in these control subjects: IgG1 (3 Ireland, 2 England, 1 Germany); IgG2 (2 Ireland, 2 Germany); IgG3 (3 Ireland, 6 Germany); and IgG4 (4 Ireland, 1 England, and 2 Germany) [21-23].

^c^Comparisons with 275 controls [21-23].

### Regressions on serum Ig levels

We performed a regression on serum IgG1 levels using these independent variables: age; sex; serum levels of IgG2, IgG3, IgG4, total IgA, and IgM; positivity for antibodies reactive with 68 kD protein, 30 kD protein, and type II collagen; and history of autoimmune manifestations. This revealed no significant associations. Corresponding regressions on serum total IgG, IgG2, IgG3, IgG4, IgA, and IgM levels also revealed no significant associations.

### Response to initial therapy of ASNHL

Initial therapy was oral corticosteroids (n = 23), intratympanic corticosteroid infusion (n = 3), oral methotrexate (n = 1), and oral cyclophosphamide (n = 1). Hearing improved significantly with initial therapy in 17 patients (60.7%; [95% CI: 40.7, 77.9]).

Response to initial therapy was observed in 14 men (77.8%) and three women (33.3%) (p = 0.0189). The mean ages of responders and non-responders were 53 ± 12 y and 53 ± 8 y, respectively (p = 0.9940). Four of 6 patients positive for anti-68 kD protein antibody (66.7%) and 13 of 22 patients negative for anti-68 kD protein antibody (59.1%) responded to initial therapy (p = 0.5610). Mean values of serum Ig levels did not differ significantly between responders and non-responders (data not shown).

We performed logistic regression on response to initial therapy using these independent variables: age; sex; serum levels of IgG1, IgG2, IgG3, IgG4, total IgA, and IgM; positivity for antibodies reactive with 68 kD protein, 30 kD protein, and type II collagen; and history of autoimmune manifestations. In an initial regression model, age, sex, and serum levels of total IgA and IgM were possible predictors of response to initial therapy (values of p ≤0.1500). In a refined model using only these four independent variables, there was a positive association of response to therapy in men (p = 0.0392) and a negative association with serum total IgA levels (p = 0.0274). This accounted for 34.6% of the model variance in the predictors of the dichotomous response variable.

### Respiratory tract infections in 28 ASNHL patients

One woman with subnormal IgG3 levels reported having increased frequency or severity of upper and lower respiratory tract infections at diagnosis of ASNHL. Her treatment included monthly IgG replacement. One man with subnormal IgG3 levels reported having recurrent upper and lower respiratory tract infections ~18 months after diagnosis of ASNHL and was subsequently treated with monthly IgG replacement.

### Prevalence of probable ASNHL in 441 patients with CVID/IgGSD

Among 406 IgGSD index patients, retrospective review of medical charts revealed evidence of ASNHL in three patients. One woman presented at age 52 years with acute bilateral hearing loss responsive to intravenous corticosteroid therapy, systemic lupus erythematosus with cerebritis, Sjögren syndrome, and subnormal serum levels of IgG3, although she was not evaluated in our clinic for these complaints. Six months later, she was referred to our clinic because she had recurrent upper and lower respiratory tract infections. She was subsequently treated with monthly IgG replacement. A 61 year-old woman with subnormal levels of IgG1 and IgG3 reported having “steroid-responsive hearing loss.” A 45 year-old man with subnormal levels of IgG1 and IgG3 was reported to have “idiopathic hearing loss responding to prednisone within three months.” Considering these patients to have probable ASNHL, our estimated prevalence of ASNHL in 406 patients with IgGSD during a mean follow-up interval of 8 ± 4 y was 0.74% [95% CI: 0.19, 2.33]. We found no reports of ASNHL in 35 index patients with CVID during the same follow-up interval (0%; [0, 12.3]).

## Discussion

A novel finding of the present study is that subnormal IgG1 and IgG3 levels are common in white adults with ASNHL. The prevalence and RR of subnormal IgG1 and IgG3 levels were significantly higher in the present patients than in 275 healthy adults in three previous reports [21-23]. Some patients with ASNHL had at diagnosis or eventually developed frequent or severe upper or lower respiratory tract infections. In index patients with CVID and IgGSD, selective or combined deficiencies of IgG1 and IgG3 and diverse autoimmune disorders are common [18-20]. In our retrospective review of the records of 441 index patients with CVID/IgGSD, we identified three other patients with probable ASNHL. Each of them had subnormal serum levels of IgG1 or IgG3, typical of other index patients with CVID/IgGSD phenotypes in this geographic area [18,30].

Our observations did not suggest a consistent chronologic relationship between reports of upper and lower respiratory tract infections and the onset of ASNHL. One of the present 28 patients presented simultaneously with the two conditions. Another patient began to experience recurrent upper and lower respiratory tract infections ~18 months after diagnosis of ASNHL. Among 406 patients with IgGSD, we identified three patients with possible ASNHL. One of them began having frequent respiratory tract infections six months after diagnosis of ASNHL. A temporal relationship between respiratory tract infections and possible ASNHL in the other two IgGSD patients could not be ascertained from available records.

There was a predominance of men in the present ASNHL cohort. Estimates from a large medical and pharmaceutical claims database revealed a slight male preponderance (male-to-female ratio 1.07:1) among persons with sudden sensorineural hearing loss. In patients 65 years of age or older, the ratio was 1.30:1 [17]. The mean age of the present 28 patients is similar to that in other ASNHL case series [2]. We observed evidence of autoimmune manifestations other than ASNHL in 21% of the present cohort, consistent with previous reports of autoimmune disorders in other persons with ASNHL [1,31,32]. Hearing improved with initial therapy in 64% of the present patients. The response rate to initial therapy with oral prednisolone treatment was 70% in 47 other patients with ASNHL [3]. Taken together, general characteristics of the 28 present patients are similar to those of patients in other ASNHL case series.

Subnormal levels of IgG1 and IgG3 in many patients with CVID or IgGSD are transmitted as dominant traits linked to human leukocyte antigen (HLA)-A and -B types and haplotypes encoded in the major histocompatibility complex on chromosome 6p [18,30]. Some HLA class II and I markers have also been associated with ASNHL [33-35]. IgG3 is the most polymorphic human IgG subclass and includes thirteen G3m allotypes (variants of Ig heavy G chains) which constitute six major G3m alleles of *IGHG3* on chromosome 14q32.33 [36]. Allotypes are characterized by differences in amino acid epitopes of the constant heavy G chains and are inherited in a Mendelian manner [36-39]. Thus, alleles that increase risk for ASNHL may be linked to genetic determinants of serum IgG1 and IgG3 levels on chromosome 6p or chromosome 14q32.33 in some patients.

Positivity for anti-68 kD protein antibody is associated with the diagnosis of autoimmune hearing loss [3,4,6,40], although no single antibody-related test result presently known establishes or excludes ASNHL diagnosis. In the present study, the percentages of the patients who had subnormal serum levels of IgG1 or IgG3 did not differ significantly between those with and those without anti-68 kD protein antibody.

Using logistic regression, we detected a positive association of response to initial therapy in men and a negative association of response to initial therapy with serum total IgA levels. In another ASNHL case series, univariable methods revealed that positivity for anti-68 kD protein antibody was a fair predictor of response to initial therapy [23]. In another report, antibody to an inner ear supporting cell antigen was significantly associated with hearing improvement after corticosteroid therapy [14]. Antibodies to recombinant choline transporter-like protein 2 were associated with responses to initial therapy in some patients [11]. Biological heterogeneity in putative target inner ear antigens and their corresponding autoantibodies across patients with ASNHL could partly explain these observations.

We observed subnormal serum levels of IgG1 or IgG3 in 46% of the present patients, although few of them reported having increased frequency or severity of respiratory tract infections at diagnosis of ASNHL. Consequently, we did not routinely quantify serum IgG specificities to capsular polysaccharides of common serotypes of *Streptococcus pneumoniae* or measure specific IgG responses to vaccination with polyvalent pneumococcal antigens or tetanus toxoid. Two of the 28 present patients developed increased frequency or severity of respiratory tract infections at the time of or after ASNHL diagnosis. Among 441 CVID/IgGSD patients, three probably had ASNHL. These observations suggest that evaluating patients with ASNHL by taking histories of infections and measuring serum Ig levels may reveal infection-related manifestations or subnormal serum levels of IgG subclasses, especially IgG1 and IgG3.

Uncertainties of the present work include the possibility that greater numbers of patients may have permitted elucidation of other relationships of available variables with subnormal serum levels of IgG1 or IgG3 or response to initial therapy. We did not determine whether initial oral or intratympanic corticosteroids or oral methotrexate or cyclophosphamide administered to alleviate hearing loss significantly affected serum Ig levels in the present patients. It is possible that other patients in the present cohort now lost to follow-up have developed either subnormal Ig levels or increased susceptibility to respiratory tract infections not present at ASNHL diagnosis. Evaluating serum Ig levels in patients with sudden hearing loss or Ménière syndrome would permit additional comparisons and contrasts among persons with hearing loss syndromes, but such evaluation was beyond the scope of the present work. Performing HLA typing or Gm allotyping or genotyping was beyond the scope of the present work.

## Conclusions

Serum levels of IgG1 or IgG3 were subnormal in 46.4% of 28 patients with ASNHL. Among adults who present with primary Ig deficiency, some may have or later develop ASNHL.

## Abbreviations

Ig: Immunoglobulin
ASNHL: Autoimmune sensorineural hearing loss
CVID: Common variable immunodeficiency
IgGSD: IgG subclass deficiency
ANA: Anti-nuclear antibody
c-ANCA: Cytoplasmic anti-neutrophil cytoplasmic antibodies
p-ANCA: Perinuclear anti-neutrophil cytoplasmic antibodies
SIEP: Serum immunoelectrophoresis
SD: Standard deviation
CI: Confidence interval
RR: Relative risk
HLA: Human leukocyte antigen
